# 534. Evaluation of a Rapid Multiplex PCR Diagnostic Device for the Detection of Microorganisms from Nasopharyngeal and Throat Swab Specimens in the Near Patient Setting

**DOI:** 10.1093/ofid/ofac492.587

**Published:** 2022-12-15

**Authors:** Jeffrey Bastar, Ashley Ky, Kristen Holmberg, Hana Bay, Daria Drobysheva, Thomas Davis, Rangaraj Selvarangan, Kathryn Doran, Laura Sifers, Daniel M Cohen, Virve Enne, Mark Steele, Cynthia Andjelic

**Affiliations:** bioMerieux, Salt Lake City, Utah; bioMérieux, Salt Lake City, Utah; bioMerieux, Salt Lake City, Utah; Biomerieux, Salt Lake City, Utah; bioMerieux, Salt Lake City, Utah; Indiana University, Indianapolis, Indiana; Children's Mercy, Leawood, Kansas; Children's Mercy Kansas City, Kansas City, Missouri; Children's Mercy Hospital, Kansas City, Missouri; Nationwide Children's Hospital, Columbus, Ohio; University College London, London, England, United Kingdom; University Health Truman Medical Center, Kansas City, Missouri; bioMerieux, Salt Lake City, Utah

## Abstract

**Background:**

Limited availability of multiplex molecular tests in the near-patient setting can impact the rapid diagnosis and treatment of patients experiencing symptoms of respiratory tract infections, including pharyngitis. The BioFire^®^ Respiratory/Sore Throat (R/ST) Panel (bioMérieux, Salt Lake City, UT), designed for use with the BioFire^®^ SpotFire System, is a PCR-based sample-to-answer diagnostic test that identifies four bacteria and 10 viruses (including SARS-CoV-2) from nasopharyngeal swab (NPS) or throat swab (TS) specimens in about 16 minutes. This study evaluated the performance of an Investigational Use Only (IUO) version of the BioFire R/ST Panel in the near patient setting.

**Methods:**

NPS and TS specimens were prospectively enrolled from symptomatic consented/assented volunteers of all ages, or obtained as residual leftover specimens. Enrollment was conducted between December 2020 and September 2021 at five study sites in the US and UK (adult and pediatric emergency departments or urgent care clinics) with testing performed by personnel representative of the intended users (non-laboratory professionals). Several analytes that were not circulating during the COVID-19 pandemic were supplemented with archived specimens of known analyte composition. Performance was determined for both sample types by comparison to FDA cleared multiplex PCR tests or culture and PCR followed by sequencing of isolates (*Streptococcus* from throat swabs).

**Results:**

A total of 1131 NPS and 452 TS prospectively collected specimens and 542 NPS and 128 TS archived specimens were tested with the BioFire R/ST Panel. For NPS specimens (prospective and archived) overall positive percent agreement (PPA) was 98.7% and negative percent agreement (NPA) was 99.1%, and for TS specimens (prospective and archived) overall PPA was 95.9% and NPA was 99.2%.

**Conclusion:**

The BioFire R/ST Panel is a sensitive, specific, and robust test for rapid detection of a wide range of organisms in NPS and TS specimens in the near-patient setting. This test is expected to aid in the timely diagnosis and appropriate management of pharyngitis and other respiratory infections.

*Data presented are from assays that have not been cleared or approved for diagnostic use.*

**Disclosures:**

**Jeffrey Bastar, PhD, MSCI**, bioMerieux: Employee|bioMerieux: Stocks/Bonds **Ashley Ky, MLS(ASCP)**, bioMérieux: Employee|bioMérieux: Stocks/Bonds **Kristen Holmberg, n/a**, bioMerieux: Employee **Hana Bay, BS**, Biomerieux: Employee|Biomerieux: Stocks/Bonds **Daria Drobysheva, PhD**, bioMerieux: Employee **Rangaraj Selvarangan, BVSc, PhD, D(ABMM), FIDSA, F(AAM)**, BioFire: Grant/Research Support|Luminex: Grant/Research Support **Virve Enne, BSc PhD**, Biomerieux: Advisor/Consultant **Cynthia Andjelic, PhD**, bioMerieux: Employee.

